# Early life stress alters microglia in ventral hippocampus and dorsal CA2 associated with anxiety-like behavior in adolescent male rats

**DOI:** 10.1007/s00221-025-07204-7

**Published:** 2025-12-08

**Authors:** Kezzia Jones, Lindsay Teliska, Martine M. Mirrione, Adrienne J. Betz

**Affiliations:** 1 Behavioral Neuroscience Program, Psychology Department, Quinnipiac University, Hamden, USA; 2 Biomedical Sciences Department, Quinnipiac University, Hamden, USA; 3 Quinnipiac University, SI-TE1, 275 Mount Carmel Ave, Connecticut 06518 Hamden, USA

**Keywords:** Microglia, Inflammation, Anxiety, Ventral hippocampus, Maternal separation, CA2, Iba1

## Abstract

Early-life stressors profoundly affect hippocampal development and function, with potentially detrimental consequences in adulthood. For example, adversity in early life increases the risk for the development of psychiatric disorders, including major depressive disorder (MDD), in adulthood. Pathological changes in the hippocampus are linked to altered microglial activity. Given the role of microglia in stress and development, we utilized a widely recognized rodent model to investigate how maternal separation influences inflammatory activity in stress-sensitive brain regions. Specifically, we examined the microglial morphology of Iba1-positive cells in the dorsal and ventral hippocampal subfields, the nucleus accumbens (NAc) core and shell, and the medial orbital and prelimbic areas of the prefrontal cortex. Our results reveal that the ventral hippocampus is vulnerable to early life stress and predicts anxiety-like behavior, as evidenced by increased amoeboid-shaped microglia. We also observed elevated amoeboid microglial morphology in the dorsal CA2 region. No significant changes in microglial morphology were observed in the NAc or prefrontal cortex between the stressed and non-stressed male adolescent rodents. These findings demonstrate microglial dysfunction in early life stress and support previous research linking stress, mood disorders, and inflammation. Targeting microglial activation and hippocampal neuroinflammation has the potential to develop novel therapeutics that will impact the rising global incidence of MDD and other mental illnesses.

## Introduction

Among psychiatric disorders, major depressive disorder (MDD) is the leading cause of disability. It is one of the most diagnosed mood disorders, according to the World Health Organization (WHO), and anxiety responses are considered a core response. MDD is a multifactorial disorder that is influenced by genetic, epigenetic, and environmental factors such as early life stress (ELS) (Kalin 2020; Kendler et al. 2003; LeMoult et al. 2020; Nestler 2014; Silva et al. 2021). Early life stress is thought to shape both the landscape of the brain and behavioral output and has long-lasting consequences. In humans, maternal neglect, war, natural disasters, sexual abuse, and/or physical abuse at an early age have been associated with the development of psychiatric mood disorders, including anxiety, schizophrenia, and MDD (Chocyk et al. 2011; Herringa et al. 2013; Lupien et al. 2009; Strathearn 2011). Further, clinical studies have indicated a mediating role of ELS hypercortisolism, as well as elevated levels of pro-inflammatory factor C-reactive protein and cytokine-mediated inflammation in adults (Bertollo et al. 2020; Liu et al. 2012; Miller and Chen 2010; Taylor et al. 2006). Other physiological abnormalities with clinical relevance from ELS include neural connectivity impairments in hippocampal-prefrontal and frontostriatal function following social isolation (Eluvathingal et al. 2006). The hippocampus, in particular, is a region involved in the psychopathologies related to ELS. ELS leads to smaller hippocampal volumes (Andersen et al. 2008; Teicher et al. 2012). In postmortem brains of patients diagnosed with MDD who experienced ELS, the dentate gyrus is smaller, and there are fewer granule neurons (Boldrini et al. 2019).

Preclinical models support these clinical findings and offer an advantage by studying the impact of ELS during developmental time periods, such as adolescence. Maternal separation in rodents has been used to investigate the neuroanatomical, neurochemical, emotional, and physiological effects of ELS (Gracia-Rubio et al. 2016; Roque et al. 2016; Vivinetto et al. 2013; Zalosnik et al. 2014). Animals exposed to maternal separation have systemic inflammation (Figueiredo et al. 2016) and brain regions such as the hippocampus (Hulshof et al. 2011; Lajud et al. 2012; Wei et al. 2015), prefrontal cortex (PFC) and the amygdala (Kim et al. 2005) The hippocampus, nucleus accumbens (NAc), and PFC appear to be most affected by maternal separation. Dendritic morphology is altered in these areas. (Monroy et al. 2010; Muhammad and Kolb 2011; Muhammad et al. 2012). In addition to anxiety-like behavior as a result of maternal separation, most studies demonstrate a reduction in overall synaptic function and cognitive impairment, whether due to neurodegeneration or loss of dendritic spines (Aisa et al. 2007; Hulshof et al. 2011). Further, evidence suggests that specific hippocampal vulnerability following maternal separation is decreased neurogenesis in the dentate gyrus (Mirescu et al. 2004; Chen et al. 2008), delayed hippocampal development, and the reduced presence of adult stem cells (Youssef et al. 2019). Others have observed changes in glial densities as a result of maternal separation stress (Chocyk et al. 2011). These findings support the maternal separation model as not only a robust model for ELS but also a role for inflammation in stress-related disorders such as MDD.

Microglia play a multi-faceted morphological and physiological role in the central nervous system, including critical functions such as phagocytosis and synaptic pruning (Geier et al. 2012; Schafer et al. 2012), cellular surveillance, release of inflammatory cytokines and chemokines (Vinet et al. 2012) neurogenesis (Butovsky et al. 2006; Gemma and Bachstetter 2013; Wakselman et al. 2008), axonal growth (Chamak et al. 1994), and neuronal development (Wei et al. 2015). Additionally, emerging evidence suggests that microglial activation may play a role in the pathogenesis of inflammation-related neurological disorders (Na et al. 2014; Solito and Sastre 2012) such as MDD. Exposure to stress in rodent models induces depressive and anxious-like behaviors and these changes have been linked to altered microglial activation (Réus et al. 2019) specifically in the hippocampus (Wang et al. 2018). Furthermore, mouse models of ELS revealed changes in phagocytic activity of microglia in the developing hippocampus (Delpech et al. 2016). While short-term microglial activity is essential for maintaining the brain environment, chronic microglial activation can have adverse effects.

Together, these clinical and preclinical findings highlight the importance of investigating microglia as a cellular contributor through which ELS exerts lasting effects. While the field of microglia research continues to update its nomenclature framework (Paolicelli et al. 2022), we considered activated microglia to be amoeboid-like and resting microglia to be ramified. Ramified microglia are considered surveillance cells (Madry et al. 2018) with extended processes emanating from the cell body. In vivo two-photon real-time imaging reveals ramified microglia constantly sampling their environment by extending and retracting their processes with little to no migration (Nimmerjahn et al. 2005) and returning to this inactive phenotype after a stress insult (Yang et al. 2021). Studies suggest a neuroprotective role for ramified microglia after microglial ablation enhanced NMDA-induced neuronal death in the hippocampus (Vinet et al. 2012). Unlike ramified microglia, amoeboid-like microglia are associated with both phagocytosis and the removal of cellular debris following injury and the release of pro-inflammatory molecules (Fu et al. 2014; Kaur and You 2000; Neumann et al. 2009). Amoeboid phenotypes have a wide globular cell body and fewer emanating processes. They also have a laminin coating that promotes pro-inflammatory phenotypes and decreases the presence of ramified or resting microglia (Sharaf et al. 2022). These findings underscore the importance of distinguishing microglial morphological phenotypes to understand better the effects of ELS on the developing adolescent brain. Here, we used ionized calcium-binding adapter molecule 1 (Iba1) as a common marker to identify microglia (Tischer et al. 2016). This highly evolutionarily conserved protein plays a crucial role in membrane ruffling, which is essential for the morphological transition from quiescent to activated microglia (Sasaki et al. 2001; Ohsawa et al. 2000).

Our studies hypothesized that male rodents exposed to ELS via maternal separation would exhibit increased levels of anxious behavior in adolescence. Additionally, we hypothesized that maternally separated animals would possess higher levels of active amoeboid-shaped microglia in brain regions sensitive to stress, particularly the ventral hippocampus. The hippocampus has a variety of afferents and projections that mediate the stress response. Substantial evidence suggests the ventral, but not the dorsal hippocampus, mediates anxiety responses (Kjelstrup et al. 2002; Bannerman et al. 2003; Padilla-Coreano et al. 2016; Parfitt et al. 2017). We hypothesized that maternal separation stress would affect Iba1 expression in the ventral hippocampus as opposed to the dorsal hippocampus. Although most work has emphasized ventral hippocampal involvement in stress and anxiety, emerging evidence suggests that the CA2 subfield may also be stress-sensitive, with distinct contributions to social and emotional regulation (Leroy et al. 2018; Piskorowski and Chevaleyre 2023; Raghuraman et al. 2023; Smith et al. 2016). In addition, CA2 pyramidal neurons show distinct dendritic integration compared to CA1, resulting in distinct patterns of information flow and corticohippocampal connectivity (Piskorowski and Chevaleyre 2012). Given the paucity of data linking CA2 to ELS, our study further examined this region to determine whether microglial alterations might represent one pathway through which maternal separation impacts hippocampal function. Additionally, we sought to determine ELS impacts on related connections by examining the PFC and nucleus accumbens (NAc). Changes in microglial morphology and overall activity may provide a possible mechanism for developmental abnormalities in the hippocampus of animals exposed to early-life stress (ELS).

## Methods

### Animals

Six pregnant female Sprague Dawley rats were obtained at embryonic day 14 (E14) from Charles River Laboratories, MA, and housed individually in a humidity-controlled vivarium at 22 °C on a 12-h light/dark cycle (06:00 h to 18:00 h). Cages were lined with corncob bedding and provided with nesting material. Animals were given food and water ad libitum. All procedures were approved by the Quinnipiac University Institutional Care and Use Committee.

### Maternal separation

**Fig. 1:**
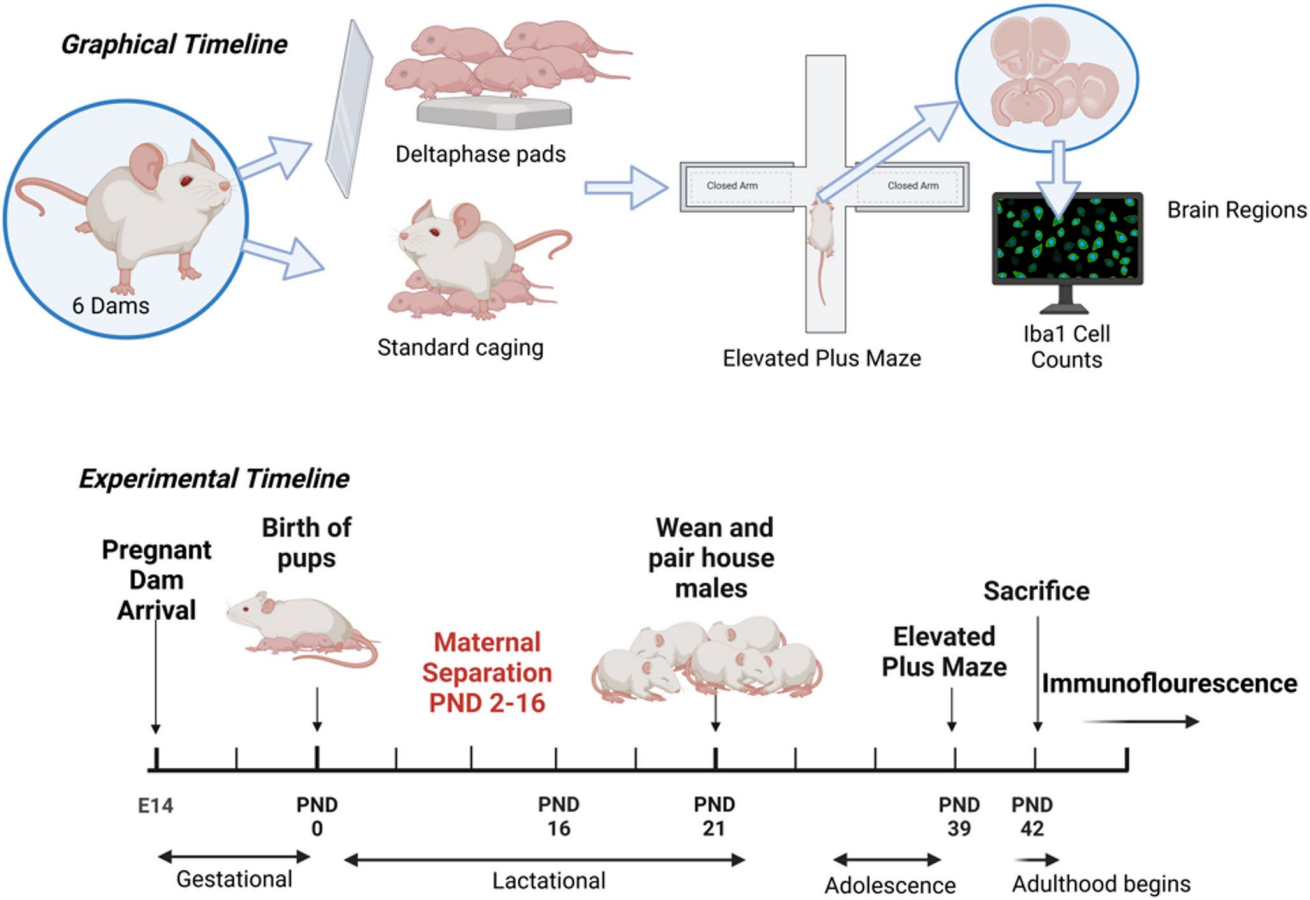
Graphical and experimental timeline for maternal separation paradigm with preparation for immunohistochemical analysis. Pregnant dams arriving at embryonic day 14 (E14) were housed and fed ad libitum for their gestational period. Newborn pups in the experimental group were separated from their dams for 3 h daily from postnatal days (PND) 2 to 16, while the control group remained with their dams and were transported outside the vivarium. All animals were returned to the vivarium after separation events. Pups were weaned on PND 21 and subjected to the elevated plus-maze as a measure of anxiety on PND 39–40. Pups from the control and experimental groups were sacrificed on PND 42, and whole brains were harvested and prepared for downstream analyses. Figure created in BioRender.com; Betz A (2025a) https://BioRender.com/t19s794

Offspring from three litters (Dams 1–3_ were assigned to the experimental group and exposed to maternal separation, while offspring from three litters (Dams 4–6) served as controls and were not exposed (n = 3 litters per group). Pups, the offspring, born between E19 and E21, were housed with their respective dams in the vivarium. Pups in the experimental group were separated from their Dams for 3 h daily while the controls remained with their dams from post-natal day (PND) 2 to 16 (See Fig. 1 for experimental schematic). For the daily separation periods, pups in the experimental group were transferred to standard rat cages without corncob bedding and placed on warm Deltaphase® Isothermal heating pads (Braintree Scientific) to maintain body temperature. The heating pads provided a constant temperature, supporting thermoregulation in the absence of the dam. Dams were placed in separate temporary holding cages. After the 3 h period, the dams and pups were returned to their original home cages. Pups in the control group remained with their dams for the duration of the experiment and therefore did not receive a Deltaphase pad. However, to control for environmental relocation, these dam-pup cages were transferred daily toanother room during daily separation periods, matching the handling and movement experienced by the experimental group. All pups were weaned on PND 21 and transferred in litters to new cages based on sex and respective dam number. Only male offspring were used in this study.

### Behavioral testing

#### Elevated plus maze

Anxiety-like behavior was measured using the elevated plus maze. Male control (n = 11) and male maternally separated (n = 11) pups were subjected to behavioral testing between PND 39 and PND 40 (Fig. 1). The maze was elevated 50 cm above the floor and consisted of four arms (10 cm wide × 50 cm long) in a plus formation. 40 cm high walls enclosed two opposing arms, and the other two arms were open. The test was performed under dim light. Pups were placed in the center of an elevated plus maze facing an open arm and allowed to explore freely for 5 min. The frequency and duration of entries into the open and closed arms were scored using Stopwatch + software (Center for Behavioral Neuroscience, Emory University) for both control and separated rats. An entry was counted when all four paws completely crossed into one arm. Evaluators were blinded to the treatment groups. The surfaces of the maze were thoroughly wiped with 70% ethanol between sessions and allowed to dry. The pups were returned to their cages in the vivarium after the behavioral tests.

### Brain sectioning

All rats were sacrificed at PND41, all rats were deeply anesthetized with CO2 and received an intracardiac perfusion of 4% paraformaldehyde solution. The whole brains were removed and suspended in a 30% sucrose solution at 4 °C. After cryoprotection, brains were washed three times for 10 min in PBS and embedded in mounting medium using Tissue-Tek O.C.T. compound (Fisher Scientific, Cat. 50–363-579), and frozen at − 80 °C for at least 15 min. Coronal sections of the entire brain were made using a Leica CM 3050 Cryostat at − 19 °C. Forty *µm* slices of free-floating sections were stored in 12-well plates loaded with a 20% glycerol, 50% ethylene glycol, 0.1 M phosphate buffer, 0.02% potassium chloride, and 0.09% sodium chloride solution at − 20 °C.

### Immunofluorescence

#### Hippocampus

Brain sections were chosen based on stereotaxic bregma coordinates (Paxinos and Watson 2007). The dorsal hippocampus (DH) corresponded to − 2.56 to − 3.00 mm, and the ventral hippocampus (VH) corresponded to − 5.40 to − 6.00 mm. A single DH and VH section within this range was chosen per animal and left and right hemispheres were analyzed. Brain tissue sections were washed three times in PBS for 10 min and blocked in 10% BSA, 20% Normal Goat Serum, and Triton X-100 in PBS for 90 min. Double staining was performed using rabbit Iba1 at 1:1000 (Wako, Cat. 019–19741) and guinea pig NeuN at 1:500 (Synaptic Systems, Cat. 266004). Brain tissues were stained in series, with Iba1 primary first, followed by NeuN, to eliminate cross-reactivity between guinea pig and rabbit hosts. After overnight incubation, tissues were washed in PBS and then incubated with goat anti-rabbit IgG H&L Alexa Fluor® 488 pre-adsorbed (Abcam, Cat. Ab150081) and goat anti-guinea pig IgG H&L Alexa Fluor® 405 (Abcam, ab175678) secondary antibodies, respectively. Brain tissues were washed in 1X PBS and mounted with 1% Gelatin solution and Fluoromount-G mounting media (Southern Biotech, Cat.0100–01).

#### Prefrontal cortex (PFC) and nucleus accumbens (NAc).

Brain sections were chosen based on stereotaxic coordinates (Paxinos and Watson 2006). The MO and PRL corresponded to bregma 2.64 mm. The NAc coordinates were 3.45 mm bregma and subdivided into the core and shell. Each section was stained as described above, with Iba1 rabbit at 1:1000 (Wako, Cat. 019–19741) and mounted with Fluoromount-G with DAPI mounting media (Southern Biotech, Cat. 0100–20) and 1% gelatin solution.

### Confocal microscopy

All microscopic images were obtained using a Nikon Eclipse E400 Confocal microscope at 20X magnification. Images were acquired in the major regions of the hippocampus, specifically the CA1, CA2, CA3, and the molecular layer of the dentate gyrus (MoDG) regions of the dorsal hippocampus. To delineate those regions, we first identified the distinctive ‘V-shaped’ crest formed by the convergence of the suprapyramidal and infrapyramidal blades of the dentate gyrus. The MoDG, a non-cellular region superficial to the suprapyramidal blade, was also used as a landmark. The densely packed pyramidal cell layer located between the blades of the dentate gyrus was considered part of the CA3 subfield. The CA2 region was distinguished CA3 by a noticeable increase in pyramidal cell layer thickness and was positioned superior to the curve of the CA3 field. CA1 was identified as a thinner pyramidal layer distal to CA2, progressing towards the subiculum. Similar strategies were employed for the the CA1, CA3, and MoDG regions for the VH. It is important to note given the paucity of data clearly segregating the CA2 region of the ventral hippocampus, this subfield was not defined or quantified in this study (Dudek et al. 2016; Insausti et al. 2023; Radzicki et al. 2023). The CA1, CA2, and CA3 regions of the dorsal hippocampus and the CA3 region of the VH were further subdivided into the Stratum Oriens (SO) and Stratum Radiatum (SR). In the PFC images were analyzed in the medial orbital (MO) and prelimbic cortex (PRL) subfields. The NAc was divided into shell and core regions for analysis. Images of the prelimbic cortex and NAc taken at 20X magnification were stitched into composite images using Microsoft Image Composite Editor 2.0.

### Image analysis and cell counting

**Fig. 2:**
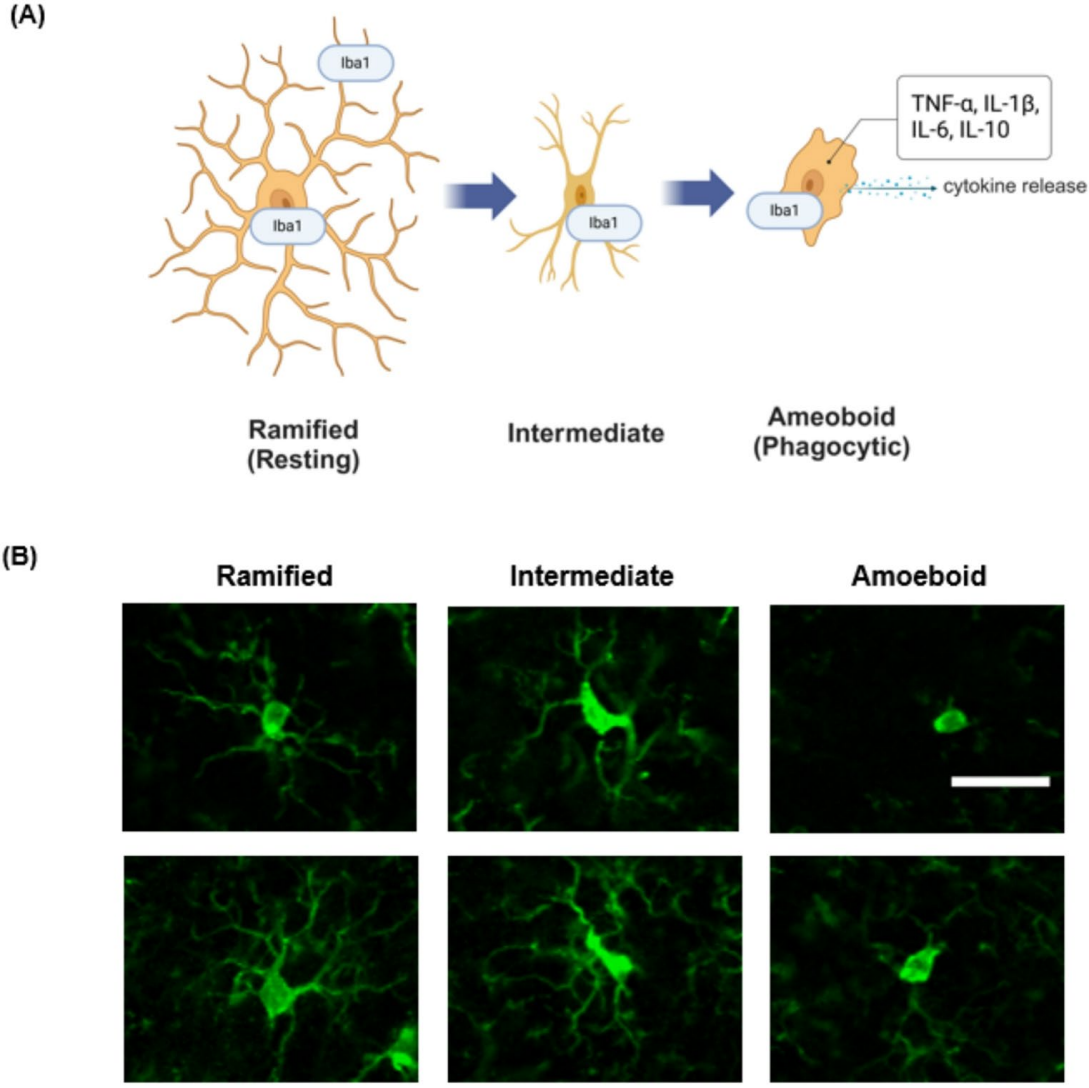
Criteria for ex vivo classification of microglial phenotypes. Immunohistochemical representations of three microglial morphological phenotypes: **A** Ramified microglia with small cell bodies and numerous thin, long processes; intermediate microglia with elongated cell bodies and thick processes indicating a transitional state; and amoeboid microglia with few short or no processes extending from the cell body. **B** The transitional phase of microglial morphology from a ramified state of surveillance and synaptic maintenance to a phagocytic amoeboid state capable of releasing pro-inflammatory cytokines such as TNF-α, IL-1β, IL-6 and IL-10 in the PFC but not hippocampus (Roque et al. 2016; Giridharan et al. 2019). Iba1 is expressed in the cytoplasm of all microglia (Korzhevskii and Kirik 2016), whether activated or quiescent. Sections were stained with Iba1, and fluorescent images were captured at 20 × magnification and processed through ImageJ to remove the background. The scale bar represents 10 µm. A portion of this figure was created in BioRender.com; Betz A (2025) https://BioRender.com/k14y577

MATLAB computing software was used to define the boundaries for each region of interest in the hippocampus and PFC. The area of each pixel was measured based on a known area (in microns) to give a conversion factor. The area calculated by MATLAB for each individual polygon was converted from pixels to mm^2^ to normalize cell counts across each region. Images from the NAc were analyzed using image J, which was calibrated for area using one standard-sized polygon for the region of interest. Based on structural morphology, Microglia were manually categorized as ramified, intermediate, or amoeboid (Fig. 2A). Iba1 staining was used to visualize microglia in each region of interest to assess the microglia type (Fig. 2B). The investigator was blinded to the treatment groups of the animals during analysis. All cell counts were independently verified with manual scoring.

### Statistical analysis

For experiments with two groups, the dependent variables were analyzed using a mixed-model two-way ANOVA and Student’s t-tests (one or two-tailed), and the results were expressed as group averages ± SEM. Post hoc comparisons were made with LSD or Tukey’s. All microglial counts were averaged between the right and left-brain sections of interest to ensure the same cell would not be counted in two sections and divided by a conversion factor to obtain microglia per mm^2^. A multiple regression analysis was performed to determine if time spent in the closed arm predicted amoeboid-like cell counts in the CA1 of the VH. Outliers (± 2 standard deviations from the mean) were excluded from analyses. Data were analyzed using SPSS and GraphPad Prism 7 and 10.3.1, and computation of effect sizes was calculated using online freeware. A p-value of less than 0.05 for all analyses was considered statistically significant.

## Results

### Maternal separation is associated with increased anxiety-like behavior in male Sprague–Dawley rats

**Fig. 3:**
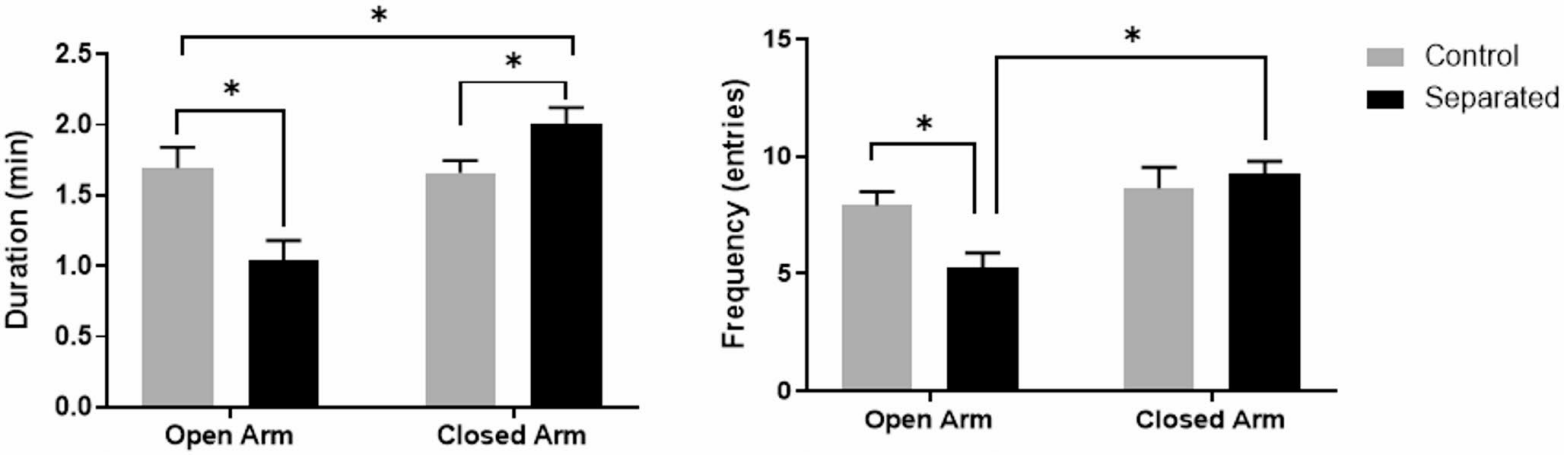
Elevated plus maze after maternal separation stress. Pups were placed in the center of an elevated plus maze for 5 min. **A** Duration and **B** frequency of entries by maternally separated (n = 11) and control rats (n = 11) into the open and closed arms of the elevated plus maze were scored using Stopwatch + software. Data are presented as mean ± SEM. * p < 0.01

The elevated plus maze is used to measure anxiety in laboratory animals. In a two-way ANOVA we determined the effects of maternal separation (versus control) and arm type (open versus closed) on time spent in the elevated plus maze. There was a main effect of treatment, F (1, 20) = 4.312, *p* = 0.05, arm type, F (1, 20) = 8.179, *p* = 0.01, and an interaction, F (1, 20) = 9.323, *p* = 0.006. Furthermore, we determined that maternally separated rats spent significantly more time (t(20) = 5.136, *p* < 0.0001), Cohen’s d = 0.992, 95% CI [0.107,1.878], indicating a large effect in the closed arm of the elevated maze compared to the open arm. Control rats showed no preference for time spent in the maze’s open or closed arm (t (20) = 0.6810, *p* = 0.5) or number of entries (t (20) = 0.1845, *p* = 0.8). Separated rats also entered the closed arm more frequently than the open arm (t (20) = 5.074, *p* < 0.0001). The overall effects for the number of entries were only found for arm type F (1, 20) = 11.92, *p* = 0.002 and an interaction of arm type and treatment F (1, 20) = 5.714, *p* = 0.02, (Fig. 3A, B).

### Ameboid microglia are increased in dorsal hippocampus CA2 but not CA1 or CA3 following maternal separation

**Fig. 4:**
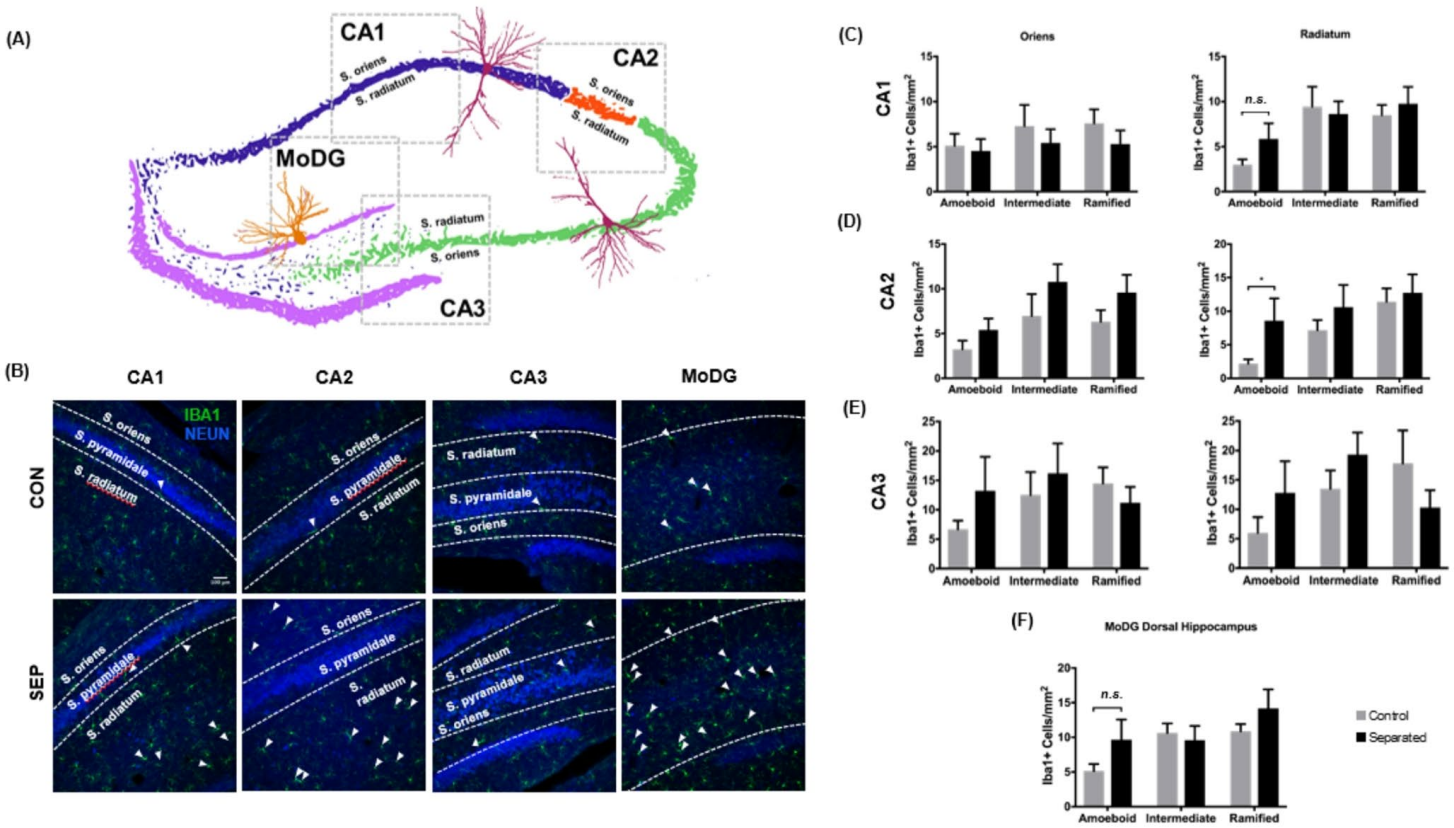
Changes in microglial morphology in the dorsal hippocampus of maternally separated rats. **A** Major sub-regions of the dorsal hippocampus at Bregma coordinate—2.64 with locations of 20 × microscope imaging designated by dashed grey boxes (sized at approximately 1mm^2^). The dentate gyrus unmyelinated granule cells called mossy fibers (pink) receive stimuli from the entorhinal cortex, which projects pyramidal neurons in the CA3 (green). Pyramidal neurons of the CA3 region form a distinctive pathway of glutamatergic connections to the CA1 (blue), with the CA2 (red) as an intermediate connection (Schaffer Collaterals). Glutamatergic neurons from the CA1 then output to the entorhinal cortex to communicate with other brain regions. Microglia cellular quantification analysis was performed for a molecular layer of the dentate gyrus (MoDG) and CA1, CA2, and CA3 in the boxed areas shown. **B** Immunofluorescent staining of the major subregions of the dorsal hippocampus in control (CON) and separated (SEP) rats. Coronal hippocampal sections were stained with Iba1 microglial marker (green) and NeuN neuronal nuclei marker (blue). Arrows indicate activated microglia in either an elongated/transitional state or a round, amoeboid shape. (**C**–**F**) Graphs showing quantification of Iba1-positive cells representative of three microglial phenotypes: amoeboid, intermediate, and ramified. Quantification of microglia with the three different morphological phenotypes in the **C** CA1 region, **D** CA2 region, **E** CA3 region and **F** molecular dentate gyrus in control (n = 9) and separated (n = 8) rats. CA1, CA2 and CA3 were subdivided into the stratum oriens (Oriens; column 1) and stratum radiatum (Radiatum; column 2). All images were captured at 20 × magnification. Data are presented as mean ± SEM. * p < 0.05. Scale bar represents 100 µm

Iba1-positive cells were quantified in the DH and subdivided into the CA1, CA2, and CA3 regions. Those regions were further subdivided into the stratum oriens, stratum radiatum, and molecular zone of the dentate gyrus (MoDG) layers (Fig. 4A). These areas differ in the number of neuronal dendrites, projections, and synapses. Brain tissue sections were stained with Iba1 and NeuN to reveal microglia and neuronal nuclei, respectively, and demonstrate variations in the activation state depending on location (Fig. 4B). In the radiatum of CA1, although there was no significant difference in amoeboid Iba1-positive cells between the control and separated groups, a significant main effect of cell morphology was observed, F(2, 44) = 6.066, *p* = 0.004. Morphological category accounted for 20.78% of the variance, while treatment group accounted for only 1.26%, suggesting that overall microglial shape differs in this region regardless of experimental condition. This contrasts with the CA1 oriens layer, where no main effects were observed (Fig. 4C). This suggests a region-specific morphological shift with the radiatum that is independent of treatment. More notably, there was an overall effect of treatment in the stratum radiatum of the CA2 DH, F (1, 45) = 3.7, *p* = 0.05. When examining just the amoeboid-like microglia in the stratum radiatum of the CA2 DH of separated rats compared to controls, we find significant differences (*t* (14) = 1.874, *p* < 0.05), Cohen’s d = 0.937, 95% CI [− 0.095,1.969], indicating a large effect(Fig. 4D). CA3 showed no significant differences between control and separated rats in the stratum radiatum or the stratum oriens for any microglial phenotypes (Fig. 4E). The MoDG layer (Fig. 4F) showed no significant change in intermediate or ramified microglia but did have an overall effect on cell morphology, F (2, 42) = 6.818, *p* = 0.002.

### Ameboid microglia are increased in ventral hippocampus CA1, MoDG, and CA3 following maternal separation

**Fig. 5:**
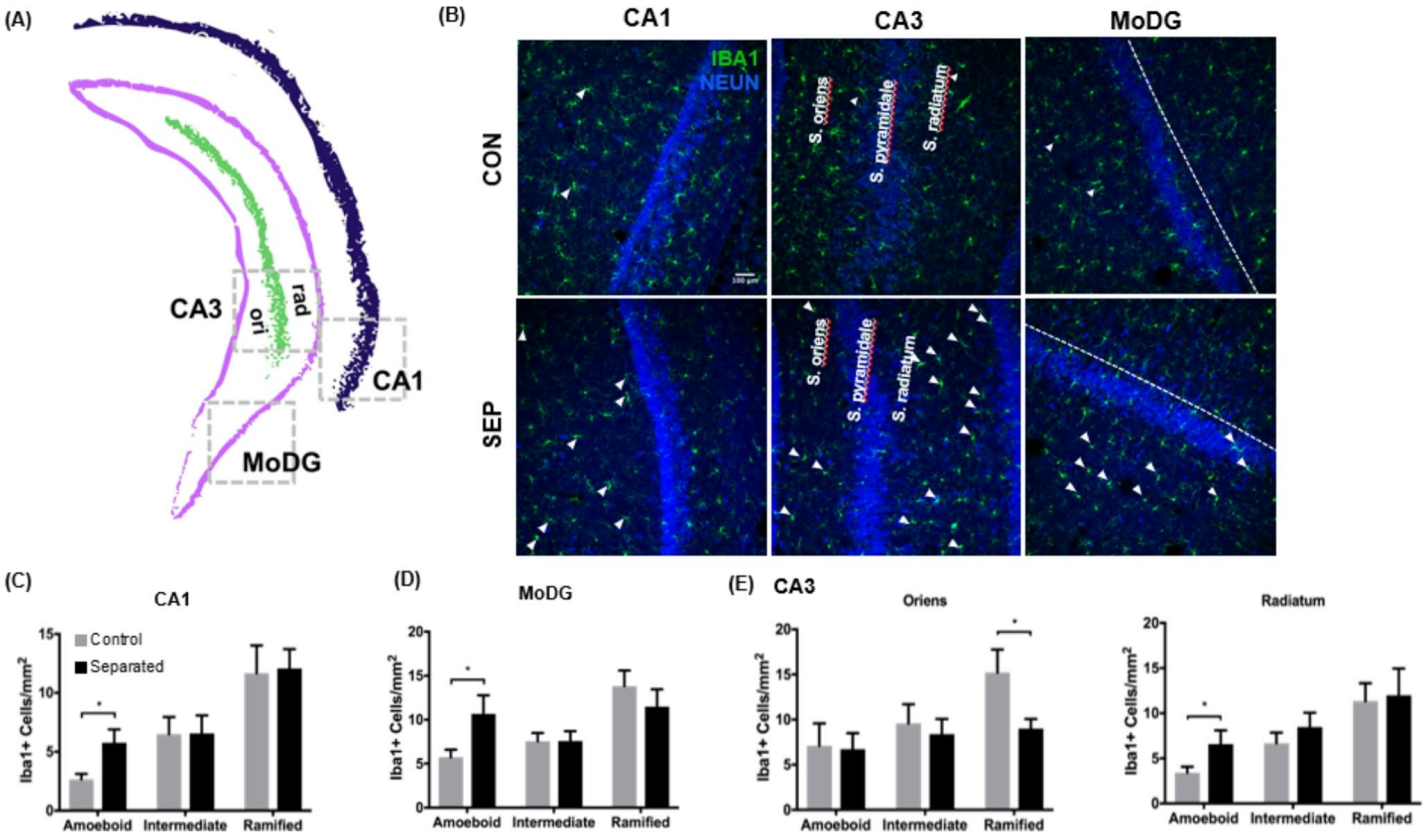
Microglial activation in the ventral hippocampus of maternally separated rats. **A** Schematic showing major sub-regions of the ventral hippocampus at Bregma coordinate -5.76 mm with locations of 20 × microscope imaging designated by dashed grey boxes (sized at approximately 1mm^2^). Dentate gyrus granule cell layer (pink), CA3 (green) and CA1 (blue) are shown. Microglia cellular quantification analysis was performed for a molecular layer of the dentate gyrus (MoDG), CA1, and CA3 in boxed areas. **B** Immunofluorescent staining of the major subregions of the ventral hippocampus in control (n = 9) and separated (n = 8) rats. Coronal hippocampal sections were stained with Iba1 microglial marker (green) and NeuN neuronal nuclei marker (blue). Arrows indicate activated microglia in either an elongated/transitional state or a round, amoeboid shape. **C**–**E** Quantification of Iba1-positive microglia in the ventral hippocampus in the CA1, molecular dentate gyrus (MoDG), and the CA3 stratum oriens and stratum radiatum sub-regions in controls and maternally separated rats. All images were captured at 20 × magnification. Data are presented as mean ± SEM. * p < 0.05. Scale bar represents 100 µm

**Fig. 6:**
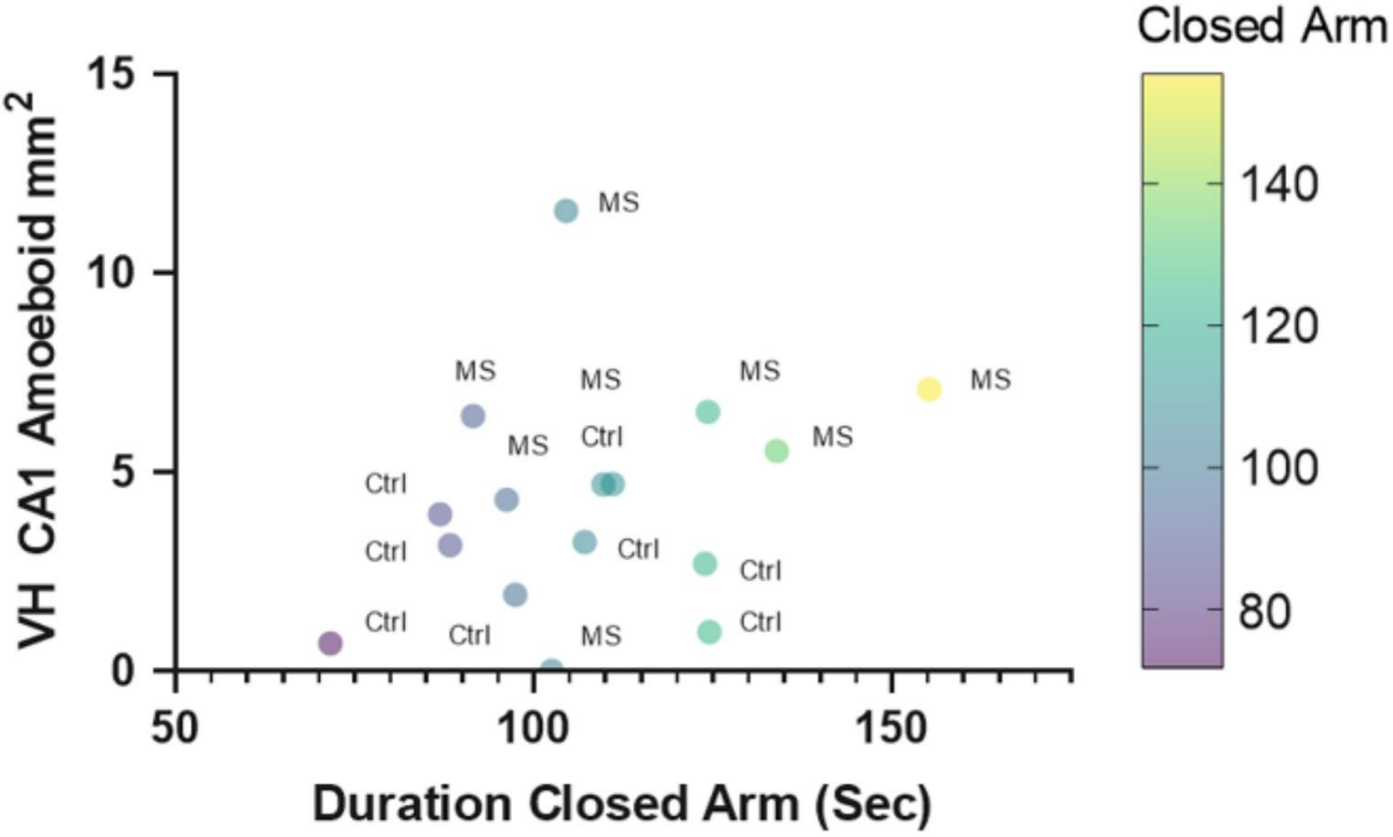
Time in closed arm of the elevated plus maze predicts amoeboid cell count in the VH. Graphical representation of time male rodents spent in the closed arm as a function of cellular counts of amoeboid-shaped microglia. Data are presented as XY coordinates and labeled with their treatment. Ctrl, control group; MS, maternally separated pups. Using multiple regression, there was a significant prediction of an increase in closed arms to the increased amount of amoeboid-shaped Iba1 cells (*p* < 0.05)

The VH was quantified as described above except the CA3 was subdivided into the stratum oriens and stratum radiatum (Fig. 5A). Subregions were again stained with Iba1 and NeuN, and variations in the activation state were demonstrated (Fig. 5B). The CA1, MoDG, and CA3 radiatum in separated rats all showed significant increases in amoeboid microglia compared to controls (t (14) = 2.492, *p* = 0.02; t (14) = 2.376, *p* = 0.03;; t (15) = 1.987, *p* = 0.03, respectively). The effect sizes and confidence intervals, respectively, are Cohen’s d = 1.246, 95% CI [0.175, 2.317]; Cohen’s d = 1.197, 95% CI [0.125, 2.268]; Cohen’s d = 0.966, 95% CI [− 041,1.972] (Fig. 5C, D, E-right panel). There was no significant difference in the number of intermediate or ramified microglia between control and separated rats in these areas. However, the CA3 stratum oriens showed a significant decline in the number of ramified microglia in separated rats, t (15) = 2.143, *p* < 0.05, Cohen’s d = 0.769, 95% CI [− 2.056,0.026] (Fig. 5E, left panel). Additionally, a multiple regression analysis was performed to examine the predictive value of the time spent in the closed arm of the elevated plus maze and amoeboid cell count in the VH CA1. The overall model was significant, R^2^ = 0.4592, F (2,13) = 5.518, *p* = 0.01. Time spent in the closed arm was a significant predictor of the number of amoeboid cells, F (1,13) = 5.375, *p* = 0.03, see Fig. 6.

### Maternal separation does not increase ameboid microglia in the NAc or PFC

**Fig. 7:**
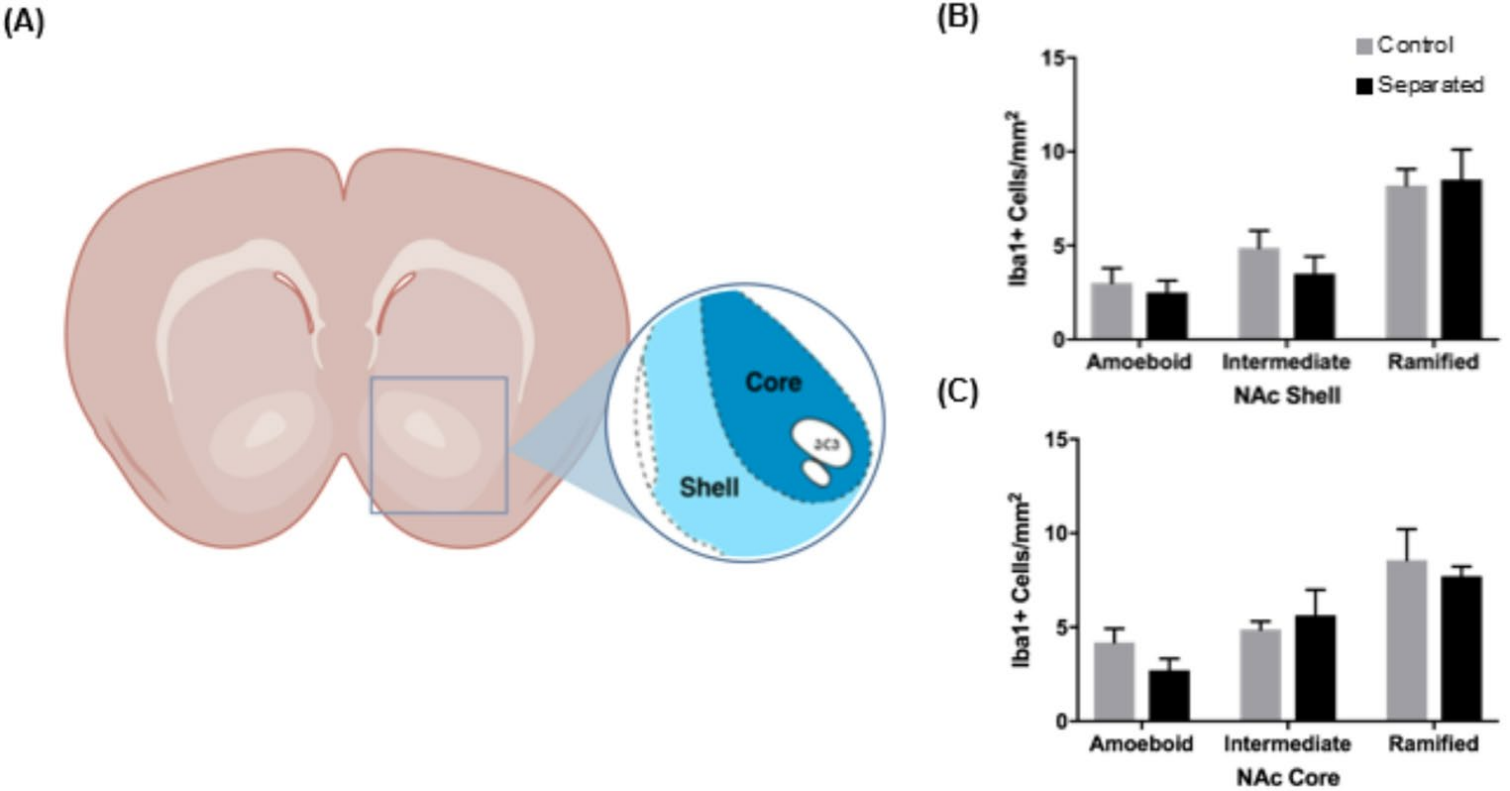
Microglial activation in the nucleus accumbens (NAc) of maternally separated rats. **A** Schematic representation of NAc (stereotaxic coordinate Bregma 3.56) adapted from Paxinos and Watson (2006). The brain tissue was stained with Iba1 (green) and DAPI (blue) and images were taken in the NAc shell and core at 20 × magnification. **B**, **C** Quantification of Iba1-positive cells representative of three microglial phenotypes: amoeboid, intermediate, and ramified. Microglia in the **B** accumbens shell and **C** core were classified based on these three different phenotypes in maternally separated (n = 8) and control (n = 8) animals. Data are presented as mean ± SEM. * p < 0.05. A portion of this figure was created in Biorender.com; Betz A (2025b) https://BioRender.com/k39r656

The NAc has connections to the hippocampus, and we analyzed both the core and shell region (Fig. 7A). There were no statistical differences between control and maternally separated cell counts for any microglia phenotype in the core of the shell of the NAc (Fig. 7B, C). In a two-way ANOVA, we determined there were differences in the quantity of each microglia phenotype and found a main effect in the shell, F (2, 42) = 16.56, and core F (2, 42) = 11.25 and post-hoc comparisons revealed a significant overall increase in amoeboid versus ramified, *p* < 0.0001 and intermediate versus ramified, *p* < 0.0005 for both subregions. This suggests a general increase in resting microglia in the NAc.

**Fig. 8:**
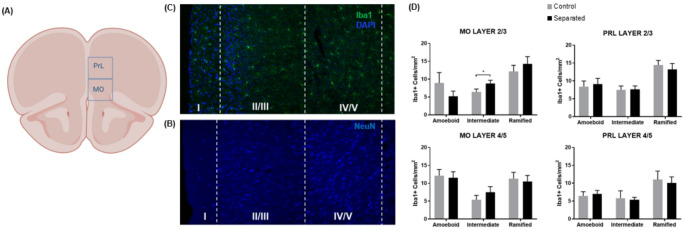
Microglial activation in the prefrontal corext (PFC) of maternally separated rats. **A** Schematic representation of mPFC containing the prelimbic (PRL) and medial orbitofrontal (MO) from Paxinos and Watson (2006) at Bregma 2.64. **B**, **C** MO and PRL cortex was stained with NeuN to show the separation of neuronal layers and Iba1 (green) and DAPI (blue). Dashed lines creating boxes represent regions’ separation of the cortical layers. All microscopic images were captured at 20 × magnification and stitched together for the composite image. **D** Quantification of three microglial phenotypes by assessment of Iba1-positive cells in the MO and PRL of controls (n = 8) and maternally separated (n = 8) rats. Data are presented as mean ± SEM. * p < 0.05. A portion of this figure was created in BioRender.com; Betz A (2025)

The PFC connects to the hippocampus, and we analyzed the MO and PRL (Fig. 8A) in distinct cortical layers (Fig. 8B and C). Quantification revealed no significant enhancement of amoeboid microglia in layers 2/3 or 4/5 (Fig. 8D) of the PFC medial orbital (MO) or prelimbic (PRL) subregions, except for significantly higher levels of intermediate microglia in the MO, t (14) = 1.841, *p* < 0.05 (Fig. 8D). In a two-way ANOVA, we determined there were differences in the quantity of each microglia phenotype and found a main effect in MO layer 2/3, F (2, 42) = 7.255 and post-hoc comparisons revealed a significant overall increase in amoeboid versus ramified, *p* < 0.0037 and intermediate versus ramified, *p* < 0.0085. A different pattern was observed for the MO layer 4/5, with a main effect of the quantity of microglia phenotype, F(2, 42) = 6.434, *p* < 0.0037. Only the comparisons of amoeboid versus intermediate and intermediate versus ramified were significant, *p* < 0.0048 and *p* < 0.02, respectively. In MO layer 4/5, there appears to be the same amount of amoeboid and ramified microglia. In contrast, in the PRL, there is a general increase in ramified microglia. There were differences in the quantity of each microglia phenotype and found a main effect in PRL layer 2/3 F (2, 42) = 11.53, *p* < 0.0001 and both amoeboid and intermediate were significantly less than ramified, *p* < 0.0021 and *p* < 0.0001, respectively. Finally, a slightly different pattern is observed in the PRL layer 4/5. Here, we see an overall effect in size type, F (2, 42) = 5.1118 *p* = 0.01 and the post hoc analysis reveals only a difference in quantity of intermediate versus ramified, *p* = 0.01. Taken together, the PFC has generally higher levels of ramified microglia independent of maternal separation treatment.

## Discussion

This study quantified Iba1-positive cells as a microglia marker to examine the link between ELS and later development of anxiety-like behavior in male rodents. This is the first study to distinguish changes in microglial activation in the hippocampal sub-regions (CA1, CA2, CA3, and MoDG) and between the ventral and dorsal hippocampus, as well as in functionally connected regions such as the PFC and NAc, of maternally separated animals. Male rats exposed to maternal separation exhibited anxiety-like behavior in the elevated plus maze, accompanied by significant changes in Iba1 amoeboid expression in specific compartments of the ventral and dorsal hippocampus but not in the PFC (MO or PRL) or NAc (core or shell).

### Maternal separation induces anxiety behavior

Several clinical and animal studies have examined the effect of ELS on development and have recognized it as a key factor dictating the onset of anxiety and depression in adulthood (Gilman et al. 2003; Nishi et al. 2013; Roque et al. 2016; Zalosnik et al. 2014). Fewer studies have examined this effect in late juvenile or early adolescence when organisms undergo significant hormonal and behavioral changes. Our study found that maternally separated male rats displayed increased anxious behavior by spending more time in the closed arm during elevated plus maze testing. While the maternal separation model has heterogeneity between studies (Wang et al. 2020) and can at times exhibit resilience under certain conditions such as pain (Genty et al. 2017) or fear responding (Macrì et al. 2011; Macrì and Würbel 2007). However, this model demonstrates markers for anxiogenic behavior and has high construct validity (Belzung and Lemoine 2011). Furthermore, it has demonstrated hypothalamic–pituitary–adrenal (HPA) axis hyperactivity observed in humans (Tyrka et al. 2008), and depressive-like behaviors persist into adulthood (Lajud et al. 2012). In rodents, the HPA axis is relatively hypo-responsive to stress during the postnatal period (2–14 days), as evidenced by low baseline levels of plasma glucocorticoids (Rosenfeld et al. 1992). Low glucocorticoid levels in the CNS are considered key for normal growth and development and are maintained by maternal care of licking and grooming. Maternal separation stress is robust enough to induce stress during this period and potentially leads to HPA hyperactivity in adulthood (Lupien et al. 2009). Unlike short-term exposure to maternal separation stress (15 min), which is thought to emulate real-life periods of maternal absence, prolonged separation (of 3 h or more a day) activates the HPA axis, elevates glucocorticoid levels, and induces anxious behavior in rodents (Lupien et al. 2009; Nishi et al. 2013; Wang et al. 2020).

In our study, animals exposed to maternal separation from PND 2 to 16 displayed anxious behavior at PND 49, characterized by a shorter time spent in open spaces and a longer time spent in close arms in the elevated plus-maze. These results align with other similar maternal separation paradigms (Aisa et al. 2007; Daniels et al. 2004; Troakes and Ingram 2009) and suggest the adolescent period is affected by ELS.

### Maternal separation differentially alters microglia activation depending on location within the hippocampus

In our study, maternally separated male rats later in adolescence exhibited higher levels of amoeboid microglia, despite having relatively similar total Iba1 counts. These results align with a study conducted by Roque et al. (2016) in which maternally separated rats showed higher levels of active microglia in the hypothalamus and hippocampus, in addition to elevated levels of cytokines (IL-6, TNF-*a* in the hypothalamus and IL-1b in the hippocampus). A shift in microglial morphology from a ramified resting state to an amoeboid state has been associated with functional change—from surveillance and resting to active phagocytosis and/or cytokine release (Ohsawa et al. 2000). Fluorescent time-lapse imaging conducted by Levtova et al. (2017) demonstrates that amoeboid microglia from human cultures were more effective at phagocytizing bioparticles, such as E. Coli, than ramified microglia. A similar time-lapse study by Stence et al. (2001) observed almost complete retraction of processes in microglial cultures 1-24 h after mechanical injury (tissue slicing), and subsequent replacement of more dynamic protrusions.

The most significant finding in our study is that microglial activation is more prominent in the VH than the DH, supporting evidence of its vulnerability to stress and inflammation (Pearson-Leary et al. 2017, 2019) and that the number of activated microglia predicts more time spent in the closed arm of the elevated plus maze, (see Fig. 6). For decades, there has been speculation about whether the ventral and dorsal hippocampus are functionally different. Extensive molecular and behavioral studies have associated the DH with processing spatial, non-episodic, and episodic memories, while the VH has been linked to emotional and affective processing (Fanselow and Dong 2010; Ferbinteanu and McDonald 2000). Additionally, lesions to the VH show reduced fear expression (Adhikari et al. 2010). Our research shows that the CA1, CA3 stratum radiatum, and MoDG regions in the VH of separated animals have higher amounts of amoeboid microglia but not intermediate or ramified microglia (see Fig. 4). The MoDG is the outermost layer of the dentate gyrus, which is comprised of granule cells and receives input from the entorhinal pathway. This area, the entrance into the hippocampus, is particularly vulnerable to stress. Development of this area occurs during the first postnatal weeks, with most granule cells generated within the first postnatal week and are origins of the adult granule cells (Muramatsu et al. 2007). The stratum radiatum is above the cell layer of CA1 and CA3 on the apical dendrite of the pyramidal neurons. In contrast, the stratum oriens is more closely aligned with the basal dendrites and axons of the pyramidal neurons or the GABA-ergic interneurons. We propose that microglial activation in the apical dendritic area is more detrimental, given its dense glutamatergic input, even in later stages of life. Patients diagnosed with Alzheimer’s disease have a significant increase in NMDA GluN1 subunit density compared to control cases in the stratum radiatum of the CA1 region (Yeung et al. 2021). Further, given the role of the microglia in synaptic sculpting (Paolicelli et al. 2011), ELS can disrupt this critical neurodevelopmental process, leading to impaired synaptic pruning and potential long-term cognitive and behavioral consequences. Taken together, both the stratum radiatum and MoDG are sensitive to ELS-induced changes in microglial activation, which may contribute to the behavioral changes we observed.

Another possible reason for the higher quantity of amoeboid-like microglia in the VH is its prominent connection to the amygdala, hypothalamus, and structures mediating neuroendocrine function. Unlike the DH, the VH has direct glutamatergic connections with the amygdala. Retrograde and anterograde amygdala studies highlight reciprocal connections confined to the CA1 subfield of the VH. However, most hippocampus-amygdala connections are inputs from the amygdala and not vice versa (Pitkänen et al. 2006). The ventral hippocampus-amygdala connection is an important factor driving fear conditioning and storing emotional memories (Felix-Ortiz and Tye 2014). Inhibition of these afferents to the VH has been shown to reduce anxiety-related behaviors, with lesions to the amygdala blocking the effect of stress on the hippocampus (Felix-Ortiz and Tye 2014; Kim et al. 2005). Additionally, a study by Jimenez et. al. (2018) highlights circuitry between the ventral hippocampus CA1 region and the lateral hypothalamus. The CA1 of the ventral hippocampus has a cluster of cells, which they refer to as “anxiety cells,” that is activated in anxiogenic environments (i.e., in the open arm of the elevated plus-maze). These cells project directly from the VH to the lateral hypothalamus but show no overlap with projections to the medial prefrontal cortex or the basal amygdala. Future studies should examine the role of microglial activation in the amygdala and hypothalamus following maternal separation.

Another important and novel finding in this study is that microglial activation is more dominant in the DH CA2 stratum radiatum layer in maternally separated male rats later in adolescence compared to control rats (see Fig. 4D). The CA2 region is a highly understudied region of the hippocampus. It is mostly considered a liaison between the CA3 pyramidal neurons and the CA1 in the trisynaptic loop, but several recent studies have highlighted its role in MDD and sociability. Recent evidence has implicated the potential role of CA2 in social memory (Hitti and Siegelbaum 2014; Stevenson and Caldwell 2014; Shivakumar et al. 2024) and social stress (Radzicki et al. 2024)**.** One important aspect of these findings is the circuit linking dorsal CA2 to the ventral CA1, an area where we had significant decreases in amoeboid cells. In several recent studies (Dang et al. 2022; Lopez-Rojas et al. 2022) it was found that cells in CA2 are selectively enhanced during social exploration and do not impair novel object recognition if silenced (Hitti and Siegelbaum 2014). Excess inflammatory activity through elevated levels of amoeboid microglia could potentially damage neurons in the CA2 and negatively influence or impair sociality in adolescence and beyond. Future studies will examine the effects of maternal separation on sociability. One explanation for a lack of ameboid activity in the stratum oriens compared to the radiatum is that the stratum oriens contains basal dendrites from pyramidal neurons, basket cells, and tri-laminar cells, collectively representing more local circuits. In contrast, retrograde and anterograde tracing have highlighted the CA2 stratum radiatum as a converging zone for projections, Schaffer collaterals, and the perforant path of humans and primates (Ding et al. 2010)**.** Taken together, this portion of the CA2 could be an integration zone for longer-range inputs, positioning itself as a key player in social memory, anxiety, and stress-related sensitivity. Our findings add to a growing body of work suggesting CA2 is important to consider as a potential target for therapeutic treatments for individuals with anxiety and MDD who have been exposed to early life stress.

### Maternal separation does not alter microglia states in the NAc and PFC

The hippocampus has projections to the NAc, which plays a role in reward/motivation and are affected in MDD (Ito et al. 2008; Vazdarjanova and Guzowski 2004). We examined the NAc because it receives glutamatergic connections from the VH, which, when stimulated, reportedly enhances susceptibility to chronic social defeat stress and induces depressive-like behavior (Bagot et al. 2015). Another study found microglial changes in these regions after social defeat stress, marked by upregulation of CD11b and decreased ramification (Wohleb et al. 2011). Surprisingly, the results of our study did not align with previous reports of microglial changes in these areas following chronic stress, although they are consistent with similar maternal separation studies. We also examined the MO and PRL of the PFC. Some studies suggest that the medial PFC has afferents directly from the CA1 region of the VH but is not directly connected to the DH (Jin and Maren 2015). Other studies suggest the MO and PRL primarily receive limbic structure afferents, including from the hippocampus and amygdala (Hoover and Vertes 2007). Banqueri et al. (2019) found that maternal separation produces a dramatic increase in astroglia cells in prefrontal areas, but Iba1 microglia cells were not increased in the prelimbic cortex (PL) and infralimbic cortex (IL). These changes could be attributed to the type of stress experienced by the animals, as well as their age and cognitive flexibility at the time of sacrifice. Most cognitive deficits are observed in adulthood, when the brain has matured, as opposed to early in life, when synaptic connections are being formed and pruned. In contrast, some studies have found that chronic stress induces microglial hyper-ramification. Although speculative, our findings align with prior work suggesting microglial morphology can be pharmacologically modulated. Hinwood et al. (2013) investigated the effects of chronic stress (restraint stress) on the prefrontal cortex by examining microglial morphology with and without the administration of a microglial inhibitor, minocycline. Their results indicate excessive branching after stress without a change in the total space occupied and subsequent decline in branching after minocycline. If we had studied cognitive flexibility or working memory or examined hyperbranching via a Scholl analysis, we might have seen the effects of ELS. Finally, we found an overall increase in ramified microglia in the superficial layers, 2/3 of the MO and PRL compared to the deeper layers, 4/5. We also found a modest increase in intermediate-type microglia in the MO layer 2/3. One interpretation is that these intermediate morphologies reflect transitional states of microglia. Majcher-Maślanka et al. (2019) found a modest decrease in Iba1-positive cells in the medial PFC and more neurons, astrocytes, and NG2-glia cells. Taken together with our results, cyELS may disrupt neuronal-glial balance, alter microglia transition states, and contribute to cortical network disorganization.

## Conclusions

Our study shows that maternal separation in male adolescent rats induces anxiety-like behavior and alters microglial morphology in the majority of subregions of the ventral hippocampus. Further, the number of amoeboid-like cells in the CA1 region of the VH predicts the time spent in the closed arm of the elevated plus maze. Elevated numbers of amoeboid-like cells are also found in the CA2 region of the dorsal hippocampus. The dorsal hippocampus appears more resilient to microglial activation associated with ELS in adolescence. While our data reveal a strong correlation between microglial morphological states and behavioral alterations following ELS, the current study does not directly establish causality. For this reason, future studies should use pharmacological or genetic tools to selectively manipulate microglia in the hippocampal subregions. In addition, incorporating automated morphometric analyses alongside manual classification could further enhance reproducibility. Furthermore, future studies utilizing this protocol should investigate microglial activity in both the juvenile period and adulthood, as well as examine sex differences. Ultimately, the CA2 region of the dorsal hippocampus may be a promising target for developing treatments for individuals with social impairments.

## Data Availability

The data supporting this study’s findings are available from the corresponding author upon reasonable request. No datasets were generated or analysed during the current study.
